# Supplementary material for: High-performance nanomaterials formed by rigid yet extensible cyclic β-peptide polymers
Source: Nat Commun. 2018 Oct 5;9:4090. doi: 10.1038/s41467-018-06576-5 (PMC6173727; doi:10.1038/s41467-018-06576-5)
Supplement: Supplementary file 1 — Supplementary Information [file 41467_2018_6576_MOESM1_ESM.pdf]

Supplementary Information

High-performance nanomaterials formed by rigid yet extensiblecyclic β-peptide polymers

Fears et al.

Supplementary Figures

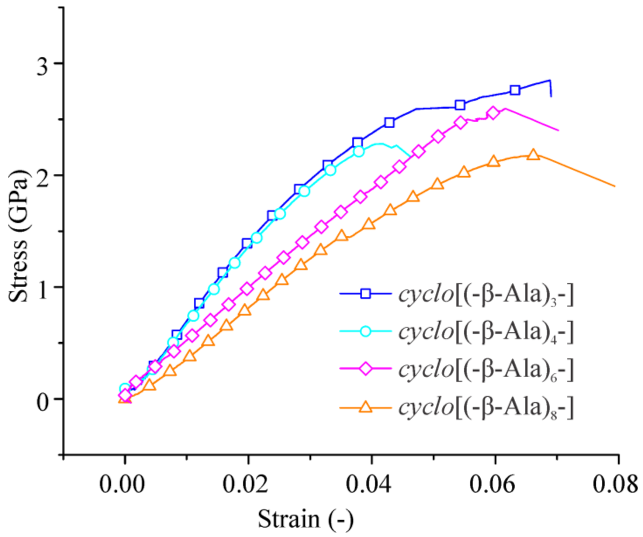

Supplementary Fig. 1. Simulated stress-strain curves for *cyclo*[(-β-Ala)$_n$-] (n=3, 4, 6, and 8).

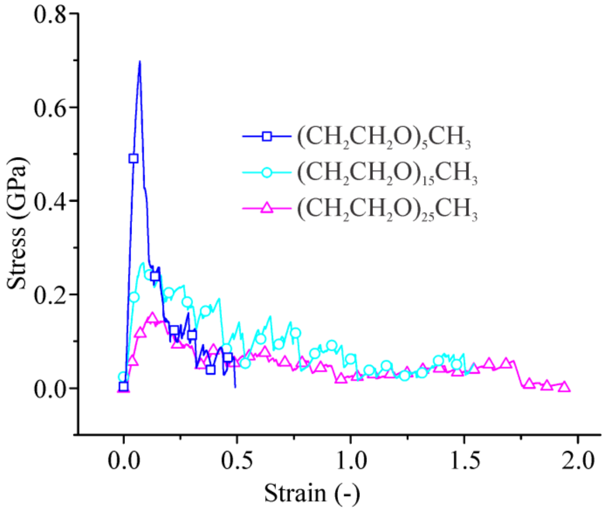

Supplementary Fig. 2. Simulated stress-strain curves for *cyclo*[(-β-HLys-β-Ala-β-HLys)-] assemblies with oligoethylene glycol grafts 5, 15, or 25 repeat units in length.

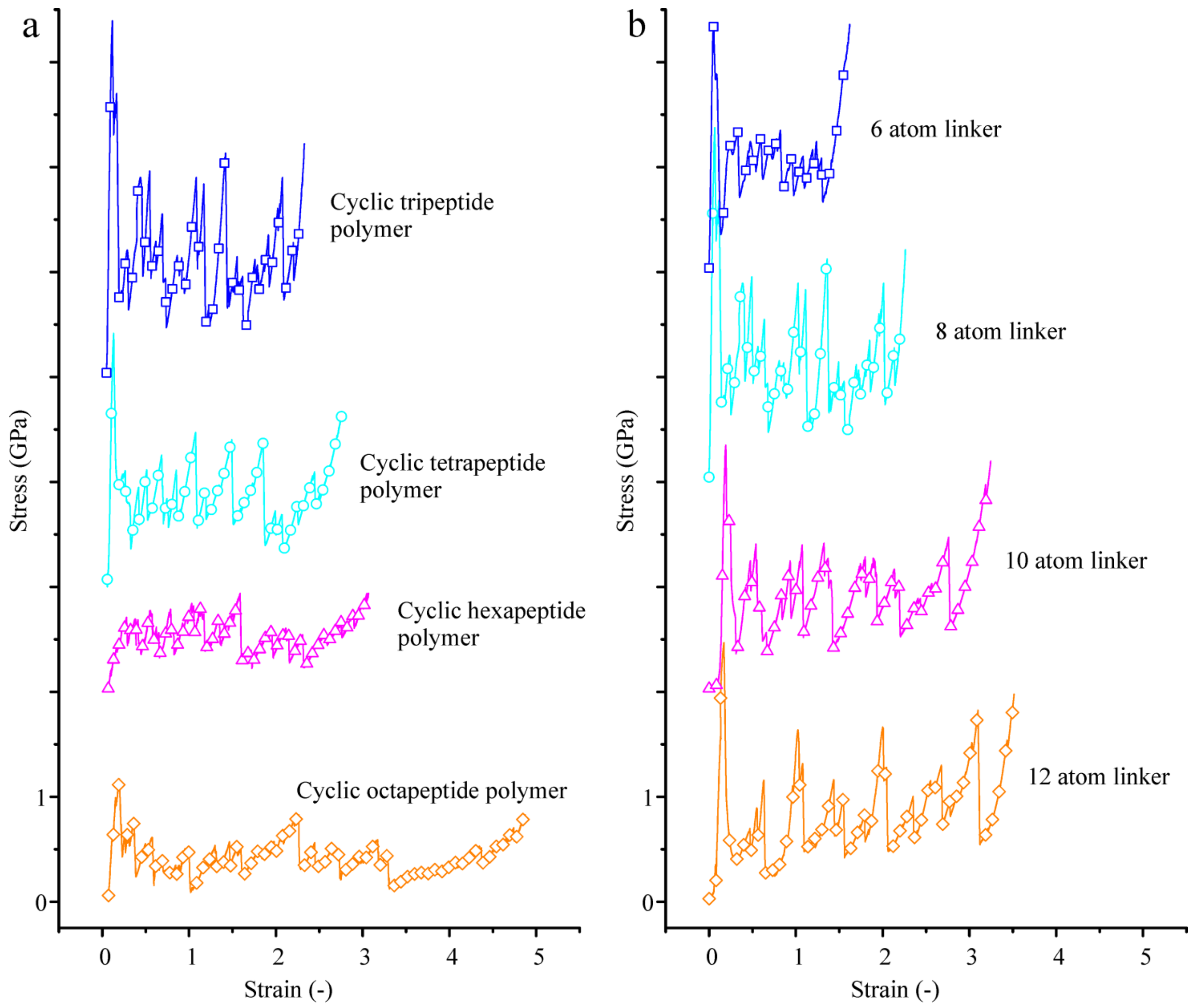

Supplementary Fig. 3. **a** Simulated stress-strain curves for a cyclic tripeptide polymer (*cyclo*[(-β-HLys-β-Ala-β-HLys)-] + *cyclo*[(-β-HGlu-β-Ala-β-HGlu)-]), a cyclic tetrapeptide polymer (*cyclo*[(-β-Ala-β-HLys-β-Ala-β-HLys)-] + *cyclo*[(-β-Ala-β-HGlu-β-Ala-β-HGlu)-]), a cyclic hexapeptide polymer (*cyclo*[(-β-Ala-β-Ala-β-HLys-β-Ala-β-Ala-β-HLys)-] + *cyclo*[(-β-Ala-β-Ala-β-HGlu-β-Ala-β-Ala-β-HGlu)-]), and a cyclic octapeptide polymer (*cyclo*[(-β-Ala-β-Ala-β-Ala-β-HLys-β-Ala-β-Ala-β-Ala-β-HLys)-] + *cyclo*[(-β-Ala-β-Ala-β-Ala-β-HGlu-β-Ala-β-Ala-β-Ala-β-HGlu)-]) with linkages between rings that are 8 atoms in length. **b** Simulated stress-strain curves for cyclic tripeptide polymers with linkages between rings that are 6, 8, 10, or 12 atoms in length.

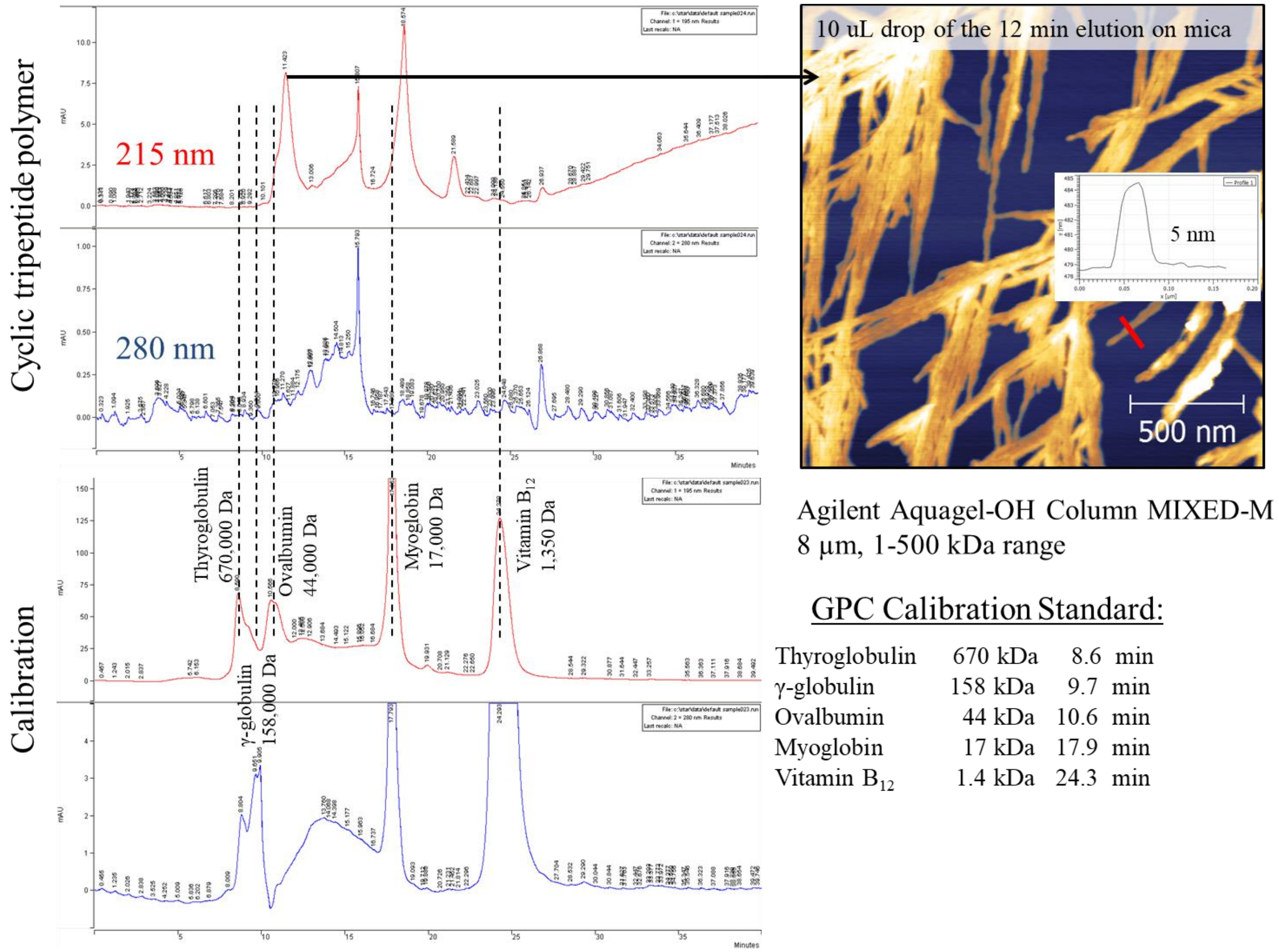

Supplementary Fig. 4. Upper chromatographs show the elution of the cyclic tripeptide polymer at 11.4 minutes. Based on the elution pattern of a protein calibration standard (lower chromatograms) the estimated molecular weight of the polymer is 40 kDa. AFM confirmed the presence of the polymer, which self-assembled into nanorod bundles after NH_4OH vapor diffusion, in the fraction that eluted at 11.4 minutes.

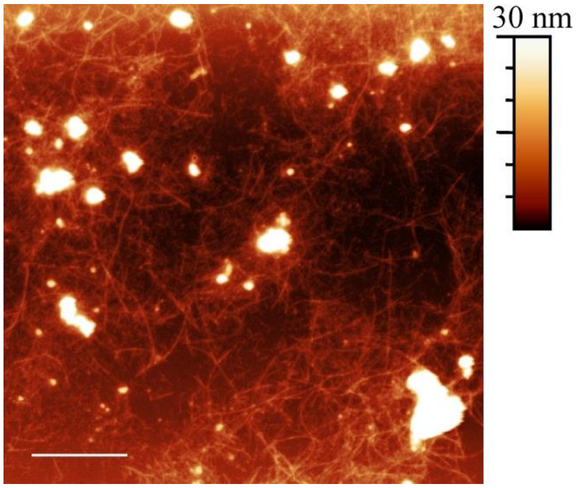

Supplementary Fig. 5. AFM image (1 μm scale bar) of PNP cast on mica from a basic PNP solution (0.33 mg mL^{-1}) in DI water 1 month after NH_4OH vapor diffusion. Color bar indicates the Z heights in the images, with the baseline (black) set to 0 nm.

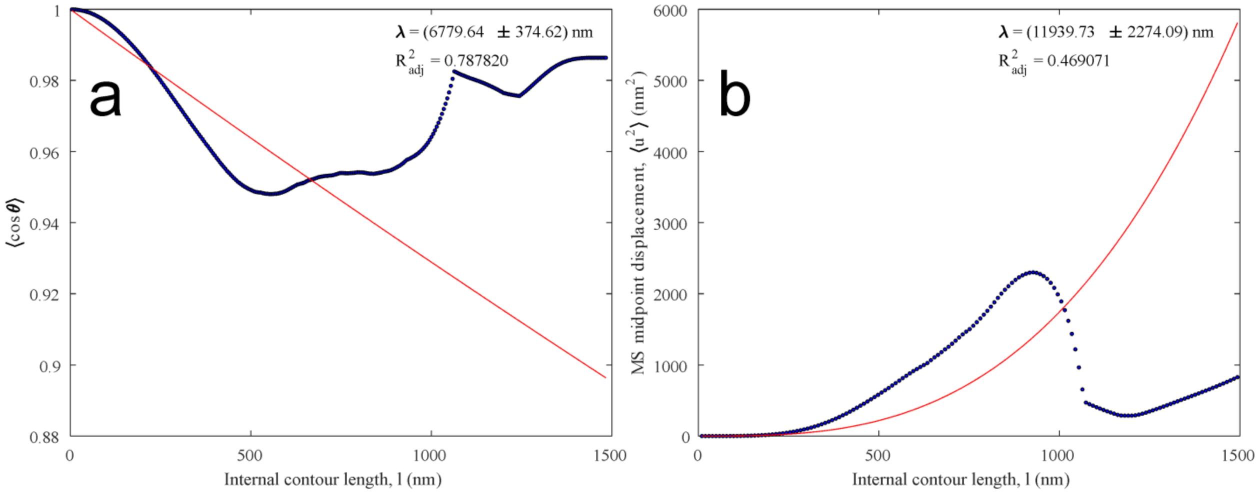

Supplementary Fig. 6. Estimation of nanorod persistence lengths calculated by fitting fluctuation in the shape of nanorods to the bond correlation function (**a**) and mean-squared midpoint displacement model (**b**). Data was acquired and analyzed using FiberApp.[15]

Supplementary Note 1

Synthesis overview

To achieve orthogonality in our synthetic scheme (vide infra), we chose to synthesize the linear precursors to **12** and **13** (Supplementary Fig. 7) via Fmoc solid-phase peptide synthesis (SPPS) using *t*-Bu-based protecting groups for β-HGlu and β-HLys side chains. For β-HOrn side chain, however, we chose 2-chlorobenzyloxycarbonyl (2-ClZ) as the protecting group due to its complete stability[1,2] toward high concentrations of trifluoroacetic acid (TFA) required to remove *t*-Bu-based protecting groups, as well as to the conditions of Fmoc SPPS. Thus, we expected that 2-ClZ groups would remain stable through synthesis and polymerization steps and could be removed by a final treatment of the protected polymer with strong acid.

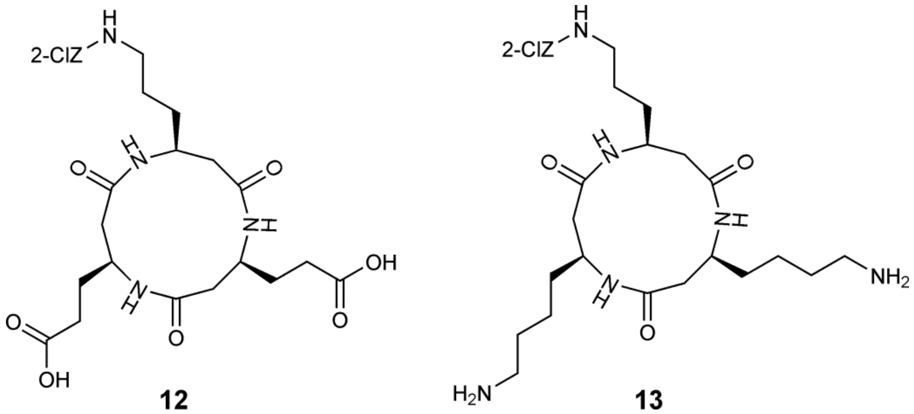

Supplementary Fig. 7. Chemical Structures of cyclic β-tripeptide subunits cyclo[β-HGlu-β-HOrn(2-ClZ)-β-HGlu] (**12**) and cyclo[β-HLys-β-HOrn(2-ClZ)-β-HLys] (**13**)

Of the three β-amino acid derivatives required for the synthesis of **12** and **13**, only Fmoc-β-HOrn(2-ClZ)-OH (**4**) was unavailable from commercial sources. We synthesized **4** via Arndt-Eistert homologation[3,4] beginning with Boc-Orn(2-ClZ)-OH **1** (Supplementary Fig. 8), since the corresponding Nα-Fmoc derivative was not commercially available. Upon obtaining Boc-β-HOrn(2-ClZ)-OH **3**, we removed Boc group with dioxane/HCl[5] and introduced an Fmoc group using Fmoc-OSu[6] to furnish **4**.

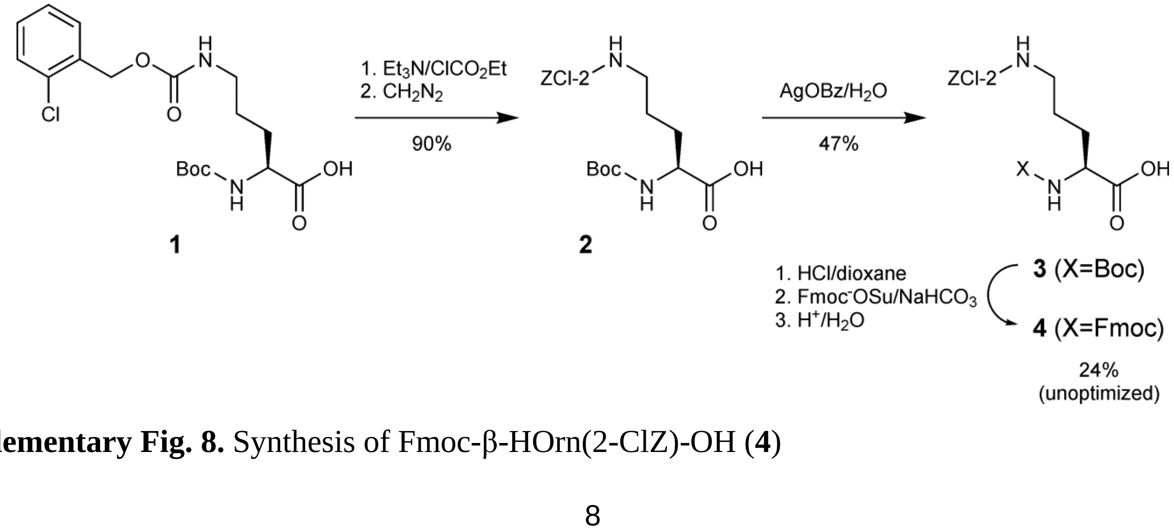

Supplementary Fig. 8. Synthesis of Fmoc-β-HOrn(2-ClZ)-OH (**4**)

We synthesized linear precursors to **12** and **13** using the highly acid-labile 2-chlorotrityl chloride resin[7] (**7**, Supplementary Fig. 9). We loaded the resin separately with Fmoc-β-HGlu(O*t*-Bu)-OH (**5**) and Fmoc-β-HLys(Boc)-OH (**6**) to give aminoacyl resins (**8** and **9**, respectively). Standard Fmoc SPPS yielded the resin-bound linear peptides (**10** and **11**, respectively). Cleavage of the peptides from the resin with 1% TFA in CH_2Cl_2 yielded the linear precursors in essentially quantitative yield, based on **8** and **9**; these gentle cleavage conditions also left the *t*-Bu-based protecting groups intact. Cyclization of the linear peptides with HATU in dimethylformamide (DMF), purification by gel-permeation chromatography, and side-chain deprotrotection with TFA/water/triisopropylsilane (TIPS) furnished **12** and **13**, both in roughly 90% overall yield.

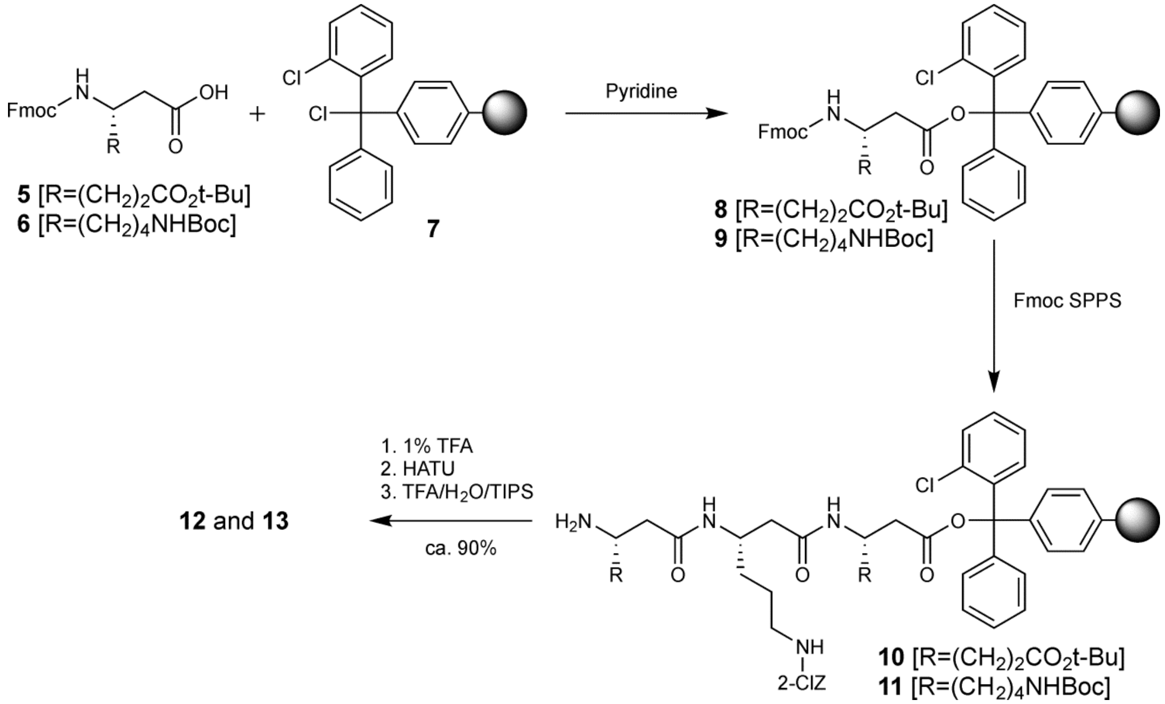

Supplementary Fig. 9. Synthesis of cyclic β-tripeptide subunits **12** and **13**

Detailed Synthesis and Characterization

Before use, THF was distilled from sodium benzophenone ketyl, while diisopropylethylamine (DIEA) was first distilled from ninhydrin and then from CaH_2.[8] Otherwise, all materials were used as received from the source indicated: Diazald®, 4 M HCl in dioxane, anhydrous Et_3N, anhydrous CH_2Cl_2, anhydrous DMF, and anhydrous pyridine, Sigma-Aldrich, Milwaukee, WI, USA; 2-chlorotrityl chloride resin, EMD Millipore, Billerica, MA, USA; N-methylpyrrolidinone (NMP, Biotech Grade, over 4 A° molecular sieves), Pharmco-Aaper, Brookfield, CT, USA; Boc-Orn(2-ClZ)-OH, Advanced ChemTech, Louisville, KY, USA; Fmoc-β-HGlu(Ot-Bu)-OH and Fmoc-β-HLys(Boc)-OH, Chem-Impex International, Wood Dale, IL, USA. Diazomethane was generated using a Mini Diazald® Apparatus with Clear-Seal® joints according to published procedures.[9]

Boc-Orn(2-ClZ) diazoketone (2). A cold soln of Boc-Orn(2-ClZ)-OH (**1**, 4.09 g, 10 mmol, 1 equiv) in dry THF (50 mL) under Ar was treated by dropwise addition of Et_3N (1.39 mL, 10 mmol, 1 equiv) followed by ClCOOEt (0.96 mL, 10 mmol, 1 equiv). The resulting mixture was stirred 15 min at -15 °C, warmed to 0 °C, then treated with an ethereal soln of diazomethane[9] (ca. 20 mmol, 2 equiv.) The reaction was allowed to reach r.t. and stirred over a period of 3 h. Excess diazomethane was destroyed by adding AcOH (ca. 3 mL), and the mixture was diluted with EtOAc (300 mL) and washed successively with sat. NH_4Cl (3×100 mL), sat. $NaHCO_3$ (4×100 mL), and brine (3×100 mL). After drying over $MgSO_4$, the solvent was removed to yield the crude product (4.12 g.) Flash chromatography on SiO_2 (30 → 45% EtOAc in hexanes) afforded **2** (Supplementary Fig. 8); yield: 3.83 g (90%); yellow solid; $[\alpha]_D^{20}$ -12.5; $[\alpha]_{436}^{20}$ -45.9; $[\alpha]_{365}^{20}$ +0.1 (c = 0.01, CH_2Cl_2).

^1H NMR (300 MHz, 330 K, 10 mM in $CDCl_3$): δ = 7.42–7.21 (m, 4 H, Ar H), 5.47 (br s, 1 H, NH), 5.22 (m, 2 H, benzylic H), 4.99 (br s, 1 H, NH), 4.20 (m, 1 H, $C^{\alpha}H$), 3.23 (m, 2 H, $C^{\delta}H_2$), 2.17, (s, 1 H, diazoketone H), 1.75 (m, 2 H, $C^{\beta}H_2$), 1.55, (m, 2 H, $C^{\gamma}H_2$), 1.53 (s, 9 H, *t*-Bu).

^{13}C NMR (75MHz, 330 K, 10 mM in $CDCl_3$): δ = 193.9, 156.5, 155.7, 134.5, 133.7, 129.9, 129.7, 129.6, 127.1, 80.3, 64.2, 56.9, 54.2, 40.6 30.0, 28.5, 26.2.

ATR-FTIR: 3355, 3095, 1703, 1673, 1627, 1536.

HRMS (ESI): *m/z* calcd for $C_{19}H_{25}N_4O_5Cl$ + Na $[M + Na]^+$: 447.1406; found: 447.1403.

Boc-β-HOrn(2-ClZ)-OH (3). Diazoketone **2** (3.52 g, 8.35 mmol, 1 equiv) and water (6.81 mL) were dissolved in THF (82 mL) at -25 °C with the exclusion of light, and silver benzoate (0.21 g, 0.91 mmol, 0.11 equiv) in Et_3N (3.30 mL, 23.86 mmol, 2.9 equiv) was added dropwise. The reaction mixture was kept at -20 °C for 3 h, then allowed to warm to r.t. over 1 h. The mixture was then diluted with EtOAc (300 mL) and washed with 0.6 M HCl (3×60 mL). After drying over $MgSO_4$, the solvent was removed to give the crude product (3.14 g). Flash chromatography on SiO_2 (0 → 5% MeOH in $CHCl_3$) afforded **3** (Supplementary Fig. 8); yield: 1.62 g (47%); white solid; $[\alpha]_D^{20}$ -21.33; $[\alpha]_{436}^{20}$ -38.0; $[\alpha]_{365}^{20}$ -65.0 (c = 0.01, CH_2Cl_2).

^1H NMR (300 MHz, 330 K, 10 mM in DMSO-d_6): δ = 7.42–7.21 (m, 4 H, Ar H), 7.08 (br s, 1 H, NH), 6.41 (br s, 1 H, NH), 5.22 (m, 2 H, benzylic H), 3.71 (m, 1 H), 2.98 (m, 2 H), 2.49 (m, 1 H), 2.31 (m, 1 H), 1.43 (m, 2 H), 1.41 (m, 2 H), 1.37 (m, 9 H, *t*-Bu).

^{13}C NMR (75MHz, 330 K, 10 mM in DMSO-d_6): δ = 172.0, 155.4, 154.7, 134.4, 131.9, 129.2, 129.1, 128.8, 126.9, 77.2, 62.2, 47.2, 41.8, 40.9, 31.5, 27.9, 25.8.

ATR-FTIR: 3472, 3332, 3061, 1722, 1690, 1659, 1574, 1533.

HRMS (ESI): *m/z* calcd for $C_{19}H_{28}N_2O_6Cl$ $[M + H]^+$: 415.1630; found: 415.1638.

Fmoc-β-HOrn(2-ClZ)-OH (4). To an ice-cooled flask containing **3** (2.43 g, 5.86 mmol, 1 equiv) was added an ice-cooled soln of HCl (4 M) in dioxane (117 mL). The resulting soln was stirred 15 min at 0

°C, then the ice bath was removed and the mixture was stirred an additional 2.25 h. The solvent was removed under reduced pressure to give an off-white tarry substance, which was triturated with Et_2O until an off-white solid was obtained. This crude hydrochloride salt was dissolved in H_2O (15 mL) containing $NaHCO_3$ (1.72 g, 20.51 mmol, 3.5 equiv). The mixture was cooled in an ice bath, and a soln of Fmoc-OSu (2.58 g, 7.64 mmol, 1.3 equiv) in dioxane (11.76 mL) was added dropwise. The mixture was stirred at 0 °C for 1 h and allowed to warm to r.t. and stirred overnight. The mixture was diluted with H_2O (100 mL) and extracted with EtOAc (2×100 mL). The combined organic extracts were back extracted with sat $NaHCO_3$, and the combined aqueous layers were acidified to pH 1 with 0.6 M HCl and extracted with EtOAc (3×100 mL). The combined EtOAc extracts were dried over $MgSO_4$ and the solvent removed under reduced pressure to furnish the crude product (2.95 g). Flash chromatography on SiO_2 (0 → 5% MeOH in $CHCl_3$) afforded **4** (Supplementary Fig. 8); yield: 0.74 g (24%); white solid; $[\alpha]_D^{20}$ +9.0; $[\alpha]_{436}^{20}$ +22.83; $[\alpha]_{365}^{20}$ +33.0 (c = 0.01, CH_2Cl_2).

^{1}H NMR (300 MHz, 330 K, 10 mM in DMSO-d_6): δ = 7.87–7.30 (m, 12 H, Ar H), 7.08 (br s, 1 H, NH), 6.99 (br s, 1 H, NH), 5.09 (s, 2 H, benzylic H), 4.29 (m, 2 H), 4.23 (m, 1 H), 3.72 (m, 1 H), 3.00 (m, 2 H), 2.48 (m, 1 H), 2.35 (m, 1 H), 1.46 (m, 2 H), 1.44 (m, 2 H).

^{13}C NMR (75MHz, 330 K, 10 mM in DMSO-d_6): δ = 172.4, 155.7, 155.5, 143.9, 143.8, 140.7, 134.6, 132.2, 129.2, 127.5, 127.2, 127.0125.1, 120.1, 65.1, 62.5, 47.8, 46.7, 41.7, 40.9, 31.6, 26.1.

ATR-FTIR: 3363, 3305, 3095, 3068, 1723, 1693, 1657, 1552.

HRMS (ESI): m/z calcd for $C_{29}H_{30}N_2O_6Cl$ $[M + H]^+$: 537.1787; found: 537.1800.

Linear peptides (lin-12) and (lin-13). A mixture of Fmoc-β-HGlu-Boc-OH **5** or Fmoc-β-HLys-Boc-OH **6** (1.56 mmol, 1.2 equiv) and 2-chlorotrityl chloride resin (**7**, 1.30 mmol, 0.98 g, 1 equiv) in dry CH_2Cl_2 (10 mL) was treated by dropwise addition of DIEA (1.03 mL, 6.24 mmol, 4.8 equiv). The mixture was stirred under argon for 2 h and was washed sequentially three times each with CH_2Cl_2/MeOH/DIEA (17:2:1), CH_2Cl_2, DMF, CH_2Cl_2, and hexanes. After drying the product overnight under vacuum, 5 mg samples were subjected to the quantitative variant of the Fmoc UV absorbance assay,[10] by which the loading of aminoacyl resins **8** and **9** was determined to be 0.43 and 0.80 mmol g^{-1}, respectively. Portions of each resin (0.29 g and 0.54 g, respectively) were carried forward at the appropriate scale (0.23 mmol).

Fmoc SPPS was carried out in DMF using 5 equiv of amino acid, 5 equiv of HATU, and 6 equiv of DIEA for each coupling reaction.[11,12] At the end of each synthesis, the N-terminal Fmoc group was removed and the resin was subjected to the usual rinsing procedure, followed by a final rinse with CH_2Cl_2. Cleavage was affected by shaking the resin for 5 min with 15 mL of 1% TFA in CH_2Cl_2. The resin was washed successively with CH_2Cl_2 (3×30 mL), MeOH (3×30 mL), CH_2Cl_2 (3×30 mL) and MeOH (3×30 mL). The rinsings were combined and the solvents were removed under reduced pressure

to yield ca. 0.20 g of crude **lin-12** and 0.20 g of crude **lin-13** (theoretical: 0.19 g and 0.21 g, respectively, for the TFA salt), which were carried forward without further purification.

Cyclic peptides (12) and (13). To a stirred soln of crude **lin-12** or **lin-13** (ca. 0.23 mmol) and HATU (0.25 g, 2.9 equiv) in anhydrous DMF (5 mL) under Ar was added DIEA (1.0 mL, 25 equiv). The resulting mixtures were stirred overnight and the solvent removed under reduced pressure to yield crude protected cyclic peptides **p-12** and **p-13**. Purification by gravity-driven gel-permeation chromatography (GPC)[13,14] on a 2.5×55 cm column of Sephadex LH-20 in degassed EtOH furnished 0.16 g of **p-12** and 0.18 g of **p-13** (ca. 100% based on aminoacyl resins **8** and **9**, respectively). The peptides exhibited the expected mass spectral characteristics: ESI-MS: *m*/*z* calcd for **p-12** $C_{34}H_{52}N_4O_9Cl$ $[M + H]^+$: 695; found 695; *m*/*z* calcd for **p-13** $C_{38}H_{62}N_6O_9Cl$ $[M + H]^+$: 781; found 781.

Removal of the *t*-Bu-based protecting groups was carried out on a 0.21 mmol scale: purified **p-12** or **p-13** was treated with excess (75 mL) cleavage cocktail (TFA/water/TIPS, 95:2.5:2.5) and stirred for 2 h. The volume was reduced to ca. 5 mL under reduced pressure and the product was precipitated with cold Et_2O to give deprotected cyclic peptides **12** and **13**; yield: ca. 90%.

***cyclo*[β-HGlu-β-HOrn(2-ClZ)-β-HGlu] (12).** ESI-MS: *m*/*z* calcd for $C_{26}H_{36}N_4O_9Cl$ $[M + H]^+$: 583; found 583; *m*/*z* calcd for $C_{26}H_{35}N_4O_9Cl$ + Na $[M + Na]^+$: 605; found: 605.

***cyclo*[β-HLys-β-HOrn(2-ClZ)-β-HLys] (13).** ESI-MS: *m*/*z* calcd for $C_{28}H_{46}N_6O_5Cl$ $[M + H]^+$: 581; found 581; *m*/*z* calcd for $C_{28}H_{45}N_5O_5Cl$ + Na $[M + Na]^+$: 603; found: 603.

Supplementary References

1 Erickson, B. W. & Merrifield, R. B. Stability of side-chain protecting groups in solid-phase peptide synthesis. *Isr. J. Chem.* **12**, 79-85, (1974).

2 Erickson, B. W. & Merrifield, R. B. Use of chlorinated benzyloxycarbonyl protecting groups to eliminate nε-branching at lysine during solid-phase peptide synthesis. *J. Am. Chem. Soc.* **95**, 3757-3763, (1973).

3 Podlech, J. & Seebach, D. On the preparation of β-amino acids from α-amino acids using the arndt-eistert reaction: Scope, limitations and stereoselectivity. Application to carbohydrate peptidation. Stereoselective α-alkylations of some β-amino acids. *Liebigs Ann.*, 1217-1228, (1995).

4 Podlech, J. & Seebach, D. The arndt-eistert reaction in peptide chemistry: A facile access to homopeptides. *Angew. Chem. Int. Edit.* **34**, 471-472, (1995).

5 Han, G., Tamaki, M. & Hruby, V. Fast, efficient and selective deprotection of the tert-butoxycarbonyl (boc) group using hcl/dioxane (4 m). *J. Pept. Res.* **58**, 338-341, (2001).

6 Lapatsanis, L., Milias, G., Froussios, K. & Kolovos, M. Synthesis of n-2,2,2-(trichloroethoxycarbonyl)-l-amino acids and n-(9-fluorenylmethoxycarbonyl)-l-amino acids involving succinimidoxy anion as a leaving group in amino-acid protection. *Synthesis*, 671-673, (1983).

7 Barlos, K. *et al.* Synthesis of protected peptide-fragments using substituted triphenylmethyl resins. *Tetrahedron Lett.* **30**, 3943-3946, (1989).

8 Carpino, L. A. & Elfaham, A. Effect of tertiary bases on o-benzotriazolyluronium salt-induced peptide segment coupling. *J. Org. Chem.* **59**, 695-698, (1994).

9 Vol. Technical Bulletin AL-180 (Sigma-Aldrich Company, St. Louis, MO, 2007).
10 Gude, M., Ryf, J. & White, P. D. An accurate method for the quantitation of fmoc-derivatized solid phase supports. *Lett. Pept. Sci.* **9**, 203-206, (2002).
11 Kulp, J. L. & Clark, T. D. Engineering a beta-helical d,l-peptide for folding in polar media. *Chem. Eur. J.* **15**, 11867-11877, (2009).
12 Clark, T. D., Sastry, M., Brown, C. & Wagner, G. Solid-phase synthesis of backbone-cyclized beta-helical peptides. *Tetrahedron* **62**, 9533-9540, (2006).
13 Vol. Handbook 18-1022-18 (Amersham Biosciencenes, Uppsala, Sweden, 2002).
14 Henke, H. *Preparative gel chromatography on sephadex lh-20*. (Huthig GmbH, 1995).
15 Usov, I. & Mezzenga, R. Fiberapp: An open-source software for tracking and analyzing polymers, filaments, biomacromolecules, and fibrous objects. *Macromolecules* **48**, 1269-1280, (2015).
